# Effects of Aerobic and Resistance Exercise Interventions on Cognitive and Physiologic Adaptations for Older Adults with Mild Cognitive Impairment: A Systematic Review and Meta-Analysis of Randomized Control Trials

**DOI:** 10.3390/ijerph17249216

**Published:** 2020-12-09

**Authors:** Junga Lee

**Affiliations:** Sports Medicine and Science, Kyung Hee University, Gyeonggi-do 17104, Korea; jalee@khu.ac.kr

**Keywords:** mild cognitive impairment, aerobic exercise, resistance exercise, randomized controlled trials, meta-analysis

## Abstract

(1) Background: The purpose of this meta-analysis is to investigate the effects of exercise intervention for older adults with mild cognitive impairment (MCI). (2) Methods: Databases including PubMed, Medline, and Cochrane were used to search for studies that reported effects of exercise for older adults with MCI and randomized controlled trials up to July 2020. Exercise interventions of all selected studies were summarized, and effect sizes of exercise interventions were calculated. (3) Results: A total of 14 studies, including 1178 older adults with MCI were included. Exercise participation in older adults with MCI improved cognitive functions (*d* = 0.88, 95% confidence interval [CI]; 0.10–1.65, *p* = 0.01; *k* = 5]) and handgrip strength (*d* = 0.62, 95% CI; 0.23–1.01, *p* = 0.00; *k* = 4) compared with control groups. Aerobic exercise or resistance exercise at moderate to vigorous levels for at least 150 min, 1 time/week, for 6 weeks was the minimum level to obtain beneficial effects from exercise for older adults with MCI. (4) Conclusions: Older adults with MCI who participated in exercise received beneficial effects, including improvement in cognitive functions and handgrip strength, but further studies to confirm the effects are needed.

## 1. Introduction

Older adults with mild cognitive impairment (MCI) have one or more cognitive impairments without a dementia diagnosis and are unable to live independently [1]. It is estimated that about 12% to 36% of older adults have MCI, and as the population of older adults increases, the prevalence of MCI will gradually increase [2,3,4]. Management of MCI determines whether older adults will have a severe cognitive impairment, including dementia, or normal cognitive functions [1]. While reduced cognitive and physical functions are a process of aging, older adults can pursue several preventive healthy behaviors, including participating in physical activity and exercise interventions, to delay aging.

There are conflicting findings regarding the effectiveness of exercise intervention for older adults with MCI [5,6]. Several previous meta-analysis studies reported that participating in exercise interventions improved cognitive function [7,8], memory function [9], and psychological outcomes [10] among older adults with MCI. Two previous meta-analyses included only randomized controlled trials [6,8], but a previous meta-analysis did not report significant positive effects on cognitive function [6]. A previous study reported participating in aerobic exercise at 76% to 85% of heart rate (HR) reserve using a treadmill, stationary bicycle, or elliptical trainer for 6 months influenced cardiorespiratory fitness, body fatness, cognition, glucose metabolism, the hypothalamic-pituitary-adrenal axis, and trophic activity [11]. In addition, participating in aerobic exercise and resistance exercise in large muscle groups that included the chest press, rowing, leg press, calves, abdominal, and lumbar at the highest load starting with 8 repetitions that increased to 10 and 12 repetitions, 2 times per week for 6 months was associated with increased cognitive functions, and physical fitness [12]. Depending on exercise type, exercise interventions were associated with cognitive and physical adaptations that included cognitive function, blood pressure, body mass index, and physical fitness [13,14,15].

For a better understanding of exercise effects among older adults with MCI, there are several studies presenting those beneficial effects depending on exercise type [9,16]. While a previous meta-analysis focused on 11 aerobic exercise studies and found favorable effects on global cognitive ability and memory among older adults [8], additional meta-analyses that include effects of resistance exercise also need to be studied as beneficial effects of aerobic exercise and resistance exercise are important for older adults with MCI. Resistance exercise was associated with increased muscle mass, which was associated with a 43% reduction in cognitive impairments in Alzheimer’s disease [17]. Thus, the purpose of this meta-analysis was to investigate the effects of exercise interventions for older adults with MCI depending on exercise type, including aerobic exercise and resistance exercise, and to evaluate suggested effective exercise intervention guidelines for older adults with MCI in randomized controlled trials.

## 2. Materials and Methods

### 2.1. Searching Processes

The Preferred Reporting Items for Systematic Review and Meta-Analysis guideline was followed for this meta-analysis [18]. Databases including EMBASE, Medline, and Cochrane were used to search relevant studies published in English from inception to June 2020. Search terms were “exercise”, “physical activity”, “mild cognitive impairment”, “memory decline”, “cognition”, and cognitive function”. All search terms were combined as possible to find all relevant studies. In addition, previously cited studies were manually added if the studies fitted the inclusion criteria. All interventions from selected studies were assessed based on the Consensus on Exercise Reporting Template (CERT): Explanation and Elaboration Statement [19]. The checklists for CERT assessment included 16 items: Reported exercise type, qualifications for training, performed individually or in a group, supervised or unsupervised, adherence to exercise, motivation strategies, decision rules for exercise progression, how the exercise progressed, description of exercise, home exercise program, non-exercise components, adverse events during exercise, exercise setting, description of exercise intervention, generic or tailored exercise, methods of tailoring, determining exercise starting level, assessments of adherence, and intervention delivery methods were evaluated.

### 2.2. Inclusion and Exclusion Criteria 

Inclusion criteria were reporting effects of exercise intervention for older adults with MCI. The exercise interventions were randomized controlled trials. Pre- and post-outcomes of selected studies in both an experimental group and a control group were provided. Exclusion criteria were not related to the effects of exercise intervention for older adults with MCI or not providing outcomes of exercise interventions. In addition, cross-sectional studies, protocol studies, and review studies were excluded. A researcher and a reviewer independently searched relevant studies based on the inclusion and exclusion criteria. When any disagreements between a researcher and a reviewer occurred during the selecting process, a further discussion was conducted to reach an agreement.

### 2.3. Statistical Analysis 

This meta-analysis was carried out using Comprehensive Meta-Analysis Version 1.25 software (Biostatic, Inc., Englewood, NJ, USA). The effect size was calculated by using the standardized mean difference statistic, which was the difference in treatment and control group means divided by the pooled standard deviation. Heterogeneity across selected studies was determined by Higgins I^2^ statistic, which determined analysis models for each outcome. If I^2^ was ≤50%, heterogeneity did not exist, thus that the analysis model used a fixed-effect model. If I^2^ was >50%, heterogeneity did exist, thus that the analysis model used a fixed-effect model. The effect sizes of selected studies were calculated if a minimum of 2 studies reported the same outcomes.

## 3. Results

**Figure 1:**
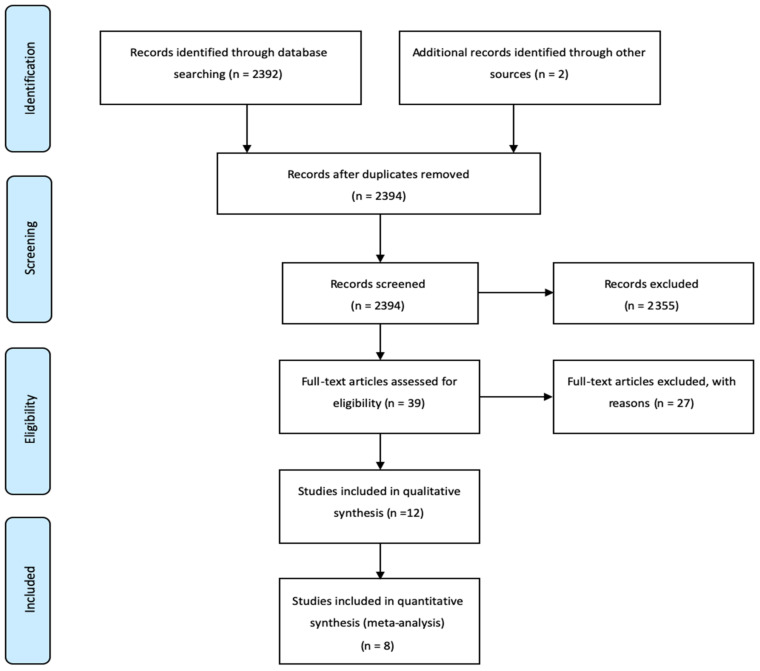
The selection process for the systematic review and meta-analysis.

Selection processes are presented in Figure 1. An initial search found 3126 studies. 3065 studies were extracted from screening titles and abstracts because the other studies did not relate to this meta-analysis or were review studies and 61 studies remained. Full texts of the remaining 61 studies were assessed to determine which were relevant. Forty-seven studies that were not randomized controlled trials, did not match mild cognitive impairment, were not exercise interventions or did not provide outcomes were excluded. Finally, 14 studies, including 1178 older adults with MCI were included [11,13,14,15,20,21,22,23,24,25,26,27,28]. The first author’s name, name of the country in which the study was conducted, year published, study design, age of participants, numbers of experimental and control groups, contents of interventions, and main findings are presented in Table 1. Older adults with MCI participated in exercise interventions for an average of 25 weeks, 3 times/week (range of average one time/week to five times/week) for 60 min, including warm-up and cool down. Two studies looked at combined exercise, ten studies at aerobic exercise, and five studies at resistance exercise at moderate to vigorous intensity. An assessment of CERT is reported in Appendix A Table A1, and the average score of 10 scores ranged from 5 to 7. Exercise type, qualifications, supervised or unsupervised, exercise progression, exercise interventions, and determination of starting levels were reported in detail in the selected studies. 

### 3.1. Effects of Exercise Interventions on Cognitive Function for Older Adults with MCI

**Figure 2:**
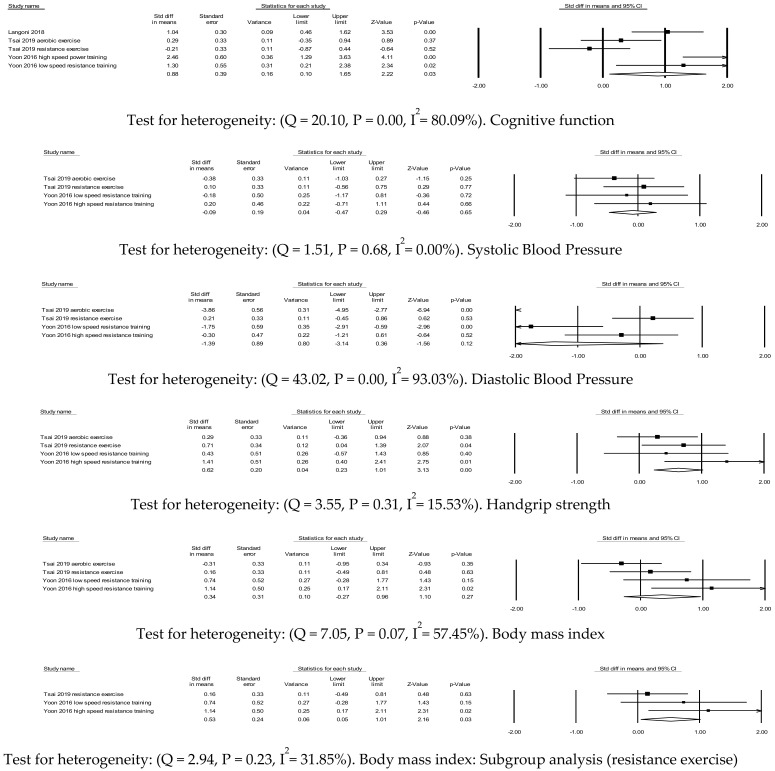
Effect sizes of exercise interventions for older adults with mild cognitive impairment.

Cognitive function was measured by the mini-mental state examination (Figure 2). Three studies including five exercise interventions (aerobic exercise or resistance exercise) were combined to calculate effect size, which was significantly large (*d* = 0.88, 95% confidence interval [CI]; 0.10–1.65, *p* = 0.01; *k* = 5]). Cognitive function was significantly increased in the exercise group compared with the control group.

### 3.2. Effects of Exercise Interventions on Blood Pressure for Older Adults with MCI

Two studies, including four exercise interventions (aerobic exercise or resistance exercise), were included for calculating effect sizes in systolic and diastolic blood pressure (Figure 2). There were no significant differences in changes in either systolic blood pressure (*d* = −0.09, 95% confidence interval [CI]; −0.47–0.29, *p* = 0.65; *k* = 4]) or diastolic blood pressure (*d* = −1.39, 95% CI; −3.14–0.36, *p* = 0.12; *k* = 4]) between the exercise group and the control group. Diastolic blood pressure had high heterogeneity, thus that the subgroup analysis was conducted depending on exercise type (aerobic exercise vs. resistance exercise), but the effect size was still not significant.

### 3.3. Effects of Exercise Interventions on Body Mass Index for Older Adults with MCI

Effect size of body mass index was not significant in the exercise group (*d* = 0.34, 95% CI; −0.27–0.96, *p* = 0.27; *k* = 4]) compared with the control group and heterogeneity was found (Figure 2). Thus, subgroup analysis was conducted depending on exercise type (aerobic exercise vs. resistance exercise). The resistance exercise intervention was only included for subgroup analysis. Effect size of body mass index was significant medium effect size (*d* = 0.53, 95% confidence interval [CI]; 0.05–1.01, *p* = 0.03; *k* = 3]) and no heterogeneity was found.

### 3.4. Effects of Exercise Interventions on Handgrip Strength for Older Adults with MCI

Effect size of handgrip strength (aerobic exercise or resistance exercise) was a significant medium effect size (*d* = 0.62, 95% confidence interval [CI]; 0.23–1.01, *p* = 0.00; *k* = 4]). Older adults with MCI in the exercise group had increased handgrip strength compared with the control group (Figure 2).

## 4. Discussion

Older adults with MCI in exercise groups had beneficial effects, including improved cognitive functions and handgrip strength, compared with control groups. No significant changes were found in blood pressure or body mass index between the exercise group and the control group. Subgroup analysis depending on exercise type and body mass index in the resistance exercise group had a significant medium effect size. The exercise groups participated in aerobic exercise or resistance exercise for an average of 25 weeks, 3 times/week for 60 min at moderate to vigorous intensity. Participating in exercise interventions may help improve the cognitive function and physical fitness of older adults with MCI.

Cognitive functions in older adults with MCI who participated in exercise interventions were improved compared with the control groups. A previous meta-analysis that reported a large effect size for exercise interventions on cognitive functions measured by the mini-mental state examination coincided with the findings of this meta-analysis [8], although the previous meta-analysis involved only aerobic exercise and this meta-analysis involved aerobic exercise and resistance exercise. Additionally, other previous meta-analysis studies found several beneficial effects on memory, delayed recall, and cognitive function that had large effect sizes associated with exercise interventions in older adults with MCI [6,9,10,29]. While diverse indicators of cognitive functions were presented in selected studies, including the verbal learning, memory test, and executive function, the effect size in this meta-analysis was calculated for the mini-mental state examination score only, which was the only possible minimal number for calculating the effect size. Findings of diverse measurements of cognitive functions and neurophysiological factors, including event-related brain potential and brain-derived neurotrophic factors, are needed in further studies.

Older adults with MCI who participated in exercise interventions increased handgrip strength compared with the control groups. The beneficial effects of exercise in older adults with MCI are meaningful because handgrip strength is a crucial predictor of physical health, sarcopenia, and overall muscle strength [30]. In addition, several previous studies reported that handgrip strength in older adults was inversely associated with cognitive functions [31,32]. Subgroup analysis in this meta-analysis found that body mass index in older adults who participated in a resistance exercise group was significantly increased compared with control groups that were cautious, while the body mass index was in the normal range (≥24.9 kg/m^2^). If lean body mass or % body fat in selected studies were provided to calculate the effect size, the reasons for increased body mass index in this meta-analysis were clear. Further studies are needed to find the effects of exercise interventions depending on body composition and exercise intervention among older adults.

Exercise interventions of selected studies in this meta-analysis were aerobic exercise or resistance exercise, for an average of 24.6 weeks, average 3 times/week, and average 1 h exercise at moderate to vigorous intensity. Ranges for the selected exercise interventions were from 6 weeks to 48 weeks. The shortest ranges, 6 weeks, walking for 30 min, 3 times/week, and Tai Chi exercise for 60 min, 3 times/week, showed increased executive function in older adults with MCI [26]. The frequency of exercise participation was from 1 time/week to 5 times/week. The shortest frequency, 1 time/week, jogging for 30 min, and shadowboxing for 60 min, also showed improved cognitive function and daily living in older adults with MCI [21]. The highest frequency, 5 times/week, cycling exercise for 30 min and resistance exercise for 30 min, showed an increase in brain-derived neurotrophic factor (BDNF) and a decrease in TNF-alpha and IL-5 [33]. Exercise intensity was estimated by reserve heat rate, maximal heart rate, or a rate of perceived exertion. Older adults with MCI may obtain a beneficial effect from participating in aerobic exercise or exercise for at least 150 min, 1 time/week, with a perceived exertion more than “somewhat hard” during 6 weeks. Based on CERT, when the selected studies were evaluated, descriptions of motivations, tailoring, adherence, and delivery methods for the interventions in the selected studies were not reported. More detailed descriptions of the processes of exercise intervention that include all lists of the CERT may help to develop more effective exercise interventions for older adults with mild cognitive impairments. Still, more studies followed by guidelines for exercise interventions that include CERT may be needed to identify the clear effects of exercise interventions in older adults with MCI. The variability of adherence to study protocols was associated with genetic factors combined with familial environmental factors [34] which need to be considered when implementing initial exercise interventions in older adults.

Several possible mechanisms of favorable effects from participating in exercise interventions can be suggested. First, participating in exercise increased BDNF in older adults with MCI who had reduced levels of BDNF precursor and mature BDNF [14,35]. These increased BDNF levels may help improve cognitive function and executive function. Second, exercise reduced levels of pro-inflammatory cytokines that are a crucial predictor of progression of MCI. Older adults with MCI also had higher levels of IL-beta, IL-6, and IL-8, but these cytokines were reduced by participating in the exercise [36,37]. Lastly, exercise in older adults with MCI increased physical fitness, including handgrip strength. This increase in physical fitness was also associated with increased BDNF and reduced inflammatory cytokines that lead positive circulations to improve cognitive functions [38].

Several limitations should be addressed. First, the number of included studies to calculate the effect size for each outcome in this meta-analysis was small, which is a limitation to the generalizability of findings. Second, while the effect sizes were calculated when the selected studies reported the same assessments and outcomes, diverse outcomes in each study could not be computed due to limited numbers of studies for calculating the effect size. Third, heterogeneity among control groups was found in the selected studies. Some control groups in the selected studies [15,20,21,22,23,26,27,28] did not participate in a physical activity intervention, but in other studies [11,12,13,24,25,33], such groups participated in some physical activity. While this meta-analysis aimed to investigate the effects of exercise interventions on older adults with MCI depending on exercise type, consistent control groups may allow us to clearly identify the effects of those interventions. Last, presentation of exercise interventions was in the form of a summary of average exercise prescriptions from each study, including exercise type, frequency, intensity, time, and period of intervention, which is a limitation due to applying the results to different body compositions, ethnicities, and sex.

## 5. Conclusions

Older adults with MCI participating in exercise interventions received positive effects, including increased cognitive functions and handgrip strength, compared with the control group. Exercise interventions were summarized as aerobic exercise or resistance exercise for an average of 3 times/week, for 60 min, for 25 weeks at moderate to vigorous intensity. Minimum suggestions for exercise participation to improve cognitive functions and physical fitness were participating in aerobic exercise or resistance exercise for at least 150 min, 1 time/week, at moderate to vigorous intensity for 6 weeks. Further studies are needed to confirm exercise effects on other outcomes such as neurophysiological effects, memory function, hemodynamic factors, and body composition.

## Figures and Tables

**Table 1:** Exercise intervention characteristics of selected studies.

First Author (year)	Design, Adherence	Participants	Exercise Intervention	Control	Major Outcomes
**Aerobic exercise**					
Baker (2010) [11]	RCT: Aerobic exercise (*n* = 19) vs. control (*n* = 10)	Exercise: Women (age 65.3 ± 9.4 years), and men (age 70.9 ± 6.7 years), and control: Women (age 74.6 ± 11.1 years) and men (age 70.6 ± 6.1 years)	24 weeks, aerobic and stretching exercise group: 4 times/week, 40 to 60 min per session, supervised exercise (the first 8 sessions, thereafter 1 session per week per participant), daily logs tracking exercise, 75% to 85% of heart rate reserve (HRR) using a treadmill, stationary bicycle, or elliptical trainer	Control group: Stretching and balance exercise, ≥50% of HR reserve exercise	Cognition, glucose metabolism, hypothalamic-pituitary-adrenal axis, trophic activity, cardiorespiratory fitness, body fat reduction, multiple tests of executive function, glucose disposal, fasting plasma levels of insulin, cortisol, brain-derived neurotrophic factor, insulin-like growth factor I, and Trails B performance
Hu (2014) [21]	RCT: Aerobic exercise (*n* = 102) vs. control (*n* = 102)	Age 70.03 ± 10.51 years	24 weeks, 1 time/week, jogging for 30 min, shadowboxing for 60 min, supervised	-	Mini-mental status examination, the activity of daily living assessment, and body movement testing
Lautenschlage (2008) [22]	RCT: Aerobic exercise (*n* = 85) vs. control (*n* = 85)	Aged 50 years or older	24 weeks, 3 times/week, 50 min of walking or strength exercise	Usual care condition	Alzheimer’s disease assessment, mood, and quality of life
Liu-Ambrose (2016) [28]	RCT: Aerobic exercise (*n* = 35) vs. control (*n* = 35)	Exercise (age 74.8 ± 8.4 years) and control (age 73.7 ± 8.3 years)	24 weeks, 3 times/week, 60 min, 60%–70% HRR, heart rate monitor, rating of perceived exertion (RPE) of 14–15	Usual care plus education	Executive interview, Alzheimer’s disease cooperative study-activities of daily living, 6-min walk distance, body mass index, and blood pressure
Scherder (2005) [26]	RCT: Aerobic exercise (*n* = 15), hand/face group (*n* = 13) vs. control (*n* = 15)	Walking (age 84 ± 6.38 years), hand/face group (age 89 ± 2.40 years), and control (age 86 ± 5.05 years)	6 weeks, walking group (30 min, 3 times/week), hand/face group (15 min, 3 times/week), Tai Chi exercise (1 h, 3 times/week)	Social visits or normal social activities	Executive functions (category naming, trail-making), memory (digit span, visual memory span, Rivermead Behavioral Memory Test, verbal learning, and memory test (direct recall, delayed recall, recognition)
Tao (2019) [23]	RCT: Aerobic exercise (*n* = 20), brisk walking (*n* = 17), vs. control (*n* = 20)	Age 60 years or older	24 weeks, 3 times/week, 60 min, 55% to 75% heart rate (Baduanjin exercise)	Did not take part in any exercise or health education	Montreal Cognitive Assessment to determine MCI, structural and functional MRI, and behavioral data analysis
ten Brinke (2015) [24]	RCT: Aerobic exercise (*n* = 24), resistance exercise (*n* = 26), vs. balance and tone exercise (*n* = 27)	Age 65 to 75 years	24 weeks, 2 times/week, 60 min, aerobic exercise (70%–80% HRR, walking), resistance exercise (6–8 repetitions, 2 sets, 7-repetition maximal (RM), biceps curls, triceps extension, seated row, latissimus dorsi pull downs, leg press, hamstring curls, and calf raises), balance and tone exercise (stretching)	Balance and tone exercise	Pulmonary function (forced expired volume), physiological measurements (VO_2peak_, iPPO, 6-min walk distance, systolic BP, diastolic BP, HRmax, HRrest), FACT-L (physical well-being, social well-being, emotional well-being, functional well-being, lung cancer subscale, trial outcome index, FACT-General, FACT-Lung), HADS (anxiety, depression), and steps
Brown (2009) [20]	RCT: Resistance exercise (*n* = 66), flexibility and relaxation (*n* = 26), vs. control (*n* = 34)	Exercise (age 75.5 ± 5.9 years), flexibility and relaxation (age 81.59 ± 6.9 years), and control (age 78.1 ± 6.4 years)	52 weeks, 5 to 15 min warm-up, 40 min conditioning (resistance training exercise, static and dynamic balance exercise, activities for challenging hand-eye and foot-eye co-ordination and flexibility, walking pattern exercise (large strides, heel-toe walking, narrow-based and wide-based walking, and sidestepping), 10 min cool-down, and flexibility and relaxation (gentle bending and rotation of the joints, trunk and neck and controlled rhythmic breathing)	Did not take part in any group activity	Medical conditions (cataracts, poor hearing, cardiovascular disease, high blood pressure, heart disease, vascular disease, diabetes, osteoarthritis, and osteoporosis), and medication use (4 + drugs, cardiovascular disease drugs, cardiovascular system drugs, psychoactive, and non-steroidal anti-inflammatory drugs)
Langoni (2018) [15]	RCT: Combined exercise (*n* = 26) vs. control (*n* = 26)	Age 60 years or older	24 weeks, 2 times/week, aerobic exercise (30 min, 60% 75 maximum heart rate) and strength exercise (30 min, 2 sets, 15 repetitions, 6 s isometric contractions, 1 min rest between sets, elbow flexion, elbow extension, external shoulder rotation, shoulder abduction, shoulder adduction, shoulder internal rotation, hip adduction, hip abduction, knee extension, knee flexion, plantar flexion, squatting, functional diagonals, knee and hip flexion-extension, hip extension, knee extension, hip abduction, hip adduction trunk flexion, plantar flexion, squatting, sit/stand from a chair	Did not take part in any exercise	Mini-mental state examination, stationary walk test, sit/stand test, and functional reach test
**Resistance exercise**					
Yoon (2017) [13]	RCT: Resistance (high speed power training, *n* = 14), resistance (low speed strength training, *n* = 9), vs. control (*n* = 7)	Age >60 years	12 weeks, 2 times/week, 1 h, high-speed power training (very low tension, a rate perceived exertion of 12–13 “somewhat hard”, 2–3 sets of 12–15 repetitions), low-speed power training (high tension, a rate perceived exertion of 15–16 “hard”, 2–3 sets of 8–10 repetitions)	Kept their routine daily activities, carried out static and dynamic stretching once/week for 1 h	Cognitive test (MMSE and MoCA-K), physical function (short physical performance battery, timed up and go test, handgrip strength), and muscle strength
**Aerobic and Resistance exercise**					
Nagamatsu (2013) [12]	RCT: Aerobic exercise (*n* = 30), resistance exercise (*n* = 28), vs. control (*n* = 28)	Age 70–80 years	24 weeks, 2 times/week, 60 min, aerobic exercise (70–80% HRR, heart rate monitor, Borg’s scale “talk”), and resistance exercise (bicep curls, triceps extension, seated row, latissimus dorsi pull downs, leg press, hamstring curls, calf raises, 7 RM, 2 sets of 6–8 repetitions	balance and tone (stretching, balance exercise, functional sand relaxation techniques)	Verbal memory and learning (Rey Auditory Verbal Learning test), and spatial memory performance
Teixeira (2018) [27]	RCT: Aerobic and resistance exercise (*n* = 20) vs. control (*n* = 20)	Age 68.3 ± 4.8 years	24 weeks, 3 times/week, aerobic exercise (70% to 90% maximum heart rate, monitoring heart rate, 30 min, walking, jogging, circuit training, balls, dancing), resistance exercise (rubber bands, basketball, volleyball, tennis, or dancing)	Did not take part in any exercise	Volume in hippocampi, memory test, functional activities, recognition, and cardiorespiratory fitness
Tsai (2019) [14]	RCT: Aerobic exercise (*n* = 15), resistance exercise (*n* = 13) vs. control (*n* = 15)	Age 60 to 85 years	16 weeks, aerobic exercise (a bicycle ergometer or a motor-driven treadmill, 70–75% of heart rate reserve (HRR), 30 min, a polar heart rate monitor), resistance exercise (75% RM, circuit exercise: bicep curls, vertical butterflies, leg presses, seated rowing, hamstring curls, calf raise, 3 sets, 10 repetitions with a 90 s rest between sets, and a 2-min interval between each apparatus)	Static stretching, balance (balance boards and fitness balls)	Event-related potential, circulating neuroprotective growth factors (BDNF, IGF-1, VEGF, FGF-2), inflammatory cytokines (TNF-α, IL-1β, IL-6, IL-8, and IL-15)
van Uffelen (2008) [25]	RCT: Aerobic (*n* = 75) vs. control (*n* = 75)	Age 70 to 80 years	52 weeks, supervised exercise, aerobic walking ( >3 metabolic equivalents)	Non-aerobic exercise, balance, flexibility, and postural exercise	Attention, and memory

## Appendix A

**Table A1:** Assessments of all interventions from selected studies based on the Consensus on Exercise Reporting Template (CERT).

Contents	Baker (2010)	Hu (2014)	Lautenschlager (2008)	Liu-Ambrose (2016)	Scherder (2005)	Tao (2019)	Ten Brink (2015)	Brown (2009)	Langoni (2018)	Yoon (2017)	Nagamatsu (2013)	Teixeira (2018)	Tsai (2019)	Van Uffelen (2008)
1. Detailed description of the type of exercise equipment	1	1	1	1	1	1	1	1	1	1	1	1	1	1
2. Detailed description of the qualifications, expertise and/or training	1	1	1	1	1	1	1	1	1	1	1	1	1	1
3. Describe whether exercises are performed individually or in a group	1	1	1	1	1	1	1	1	1	1	1	1	1	1
4. Describe whether exercises are supervised or unsupervised; how they are delivered	1	1	1	1	1	1	1	1	1	1	1	1	1	1
5. Detailed description of how adherence to exercise is measured and reported	0	0	0	0	0	0	0	0	0	0	0	0	0	0
6. Detailed description of motivation strategies	0	0	0	0	0	0	0	0	0	0	0	0	0	0
7. Detailed description of the decision rule(s) for determining exercise progression	1	0	0	0	0	0	0	0	0	0	0	0	0	0
7a. Detailed description of how the exercise program was progressed	0	0	0	0	0	0	0	0	0	0	0	0	0	0
8. Detailed description of each exercise to enable replication	1	1	1	1	1	1	1	1	1	1	1	1	1	1
9. Detailed description of any home program component	0	0	0	0	0	0	0	0	0	0	0	0	0	0
10. Describe whether there are any non-exercise components	0	0	0	0	0	0	0	0	0	0	0	0	0	0
11. Describe the type and number of adverse events that occur during exercise	1	1	1	1	1	1	1	1	1	1	1	1	1	1
12. Describe the setting in which the exercises are performed	1	1	1	1	1	1	1	1	1	1	1	1	1	1
13. Detailed description of the exercise intervention	1	1	1	1	1	1	1	1	1	1	1	1	1	1
14a. Describe whether the exercises are generic (one size fits all) or tailored	1	1	1	1	1	1	1	1	1	1	1	1	1	1
14b. Detailed description of how exercises are tailored to the individual	0	0	0	0	0	0	0	0	0	0	0	0	0	0
15. Describe the decision rule for determining the starting level	1	1	1	1	1	1	1	1	1	1	1	1	1	1
16a. Describe how adherence or fidelity is assessed/measured	0	0	0	0	0	0	0	0	0	0	0	0	0	0
16b. Describe the extent to which the intervention was delivered as planned	0	0	0	0	0	0	0	0	0	0	0	0	0	0
